# Bundled HIV and Hepatitis C Testing in the Emergency Department: A Randomized Controlled Trial

**DOI:** 10.5811/westjem.2018.8.37827

**Published:** 2018-09-10

**Authors:** Ethan Cowan, Heather S. Herman, Sara Rahman, Jennifer Zahn, Jason Leider, Yvette Calderon

**Affiliations:** * Mount Sinai Beth Israel, Department of Emergency Medicine, New York, New York; † Cornell University, Division of Nutritional Sciences, Ithaca, New York; ‡ Jacobi Medical Center, Department of Internal Medicine, Bronx, New York

## Abstract

**Introduction:**

An estimated 25% of the 1.2 million individuals living with human immunodeficiency virus (HIV) in the U.S. are co-infected with hepatitis C (HCV). The Centers for Disease Control and Prevention recommends HCV testing for high-risk groups. Our goal was to measure the impact of bundled HIV and HCV testing vs. HIV testing alone on test acceptance and identification of HCV and HIV.

**Methods:**

We conducted a two-armed, randomized controlled trial on a convenience sample of 478 adult patients in the Jacobi Medical Center emergency department from December 2012 to May 2013. Participants were randomized to receive either an offer of bundled HIV/HCV testing or HIV testing alone. We compared the primary outcome, HIV test acceptance, between the two groups. Secondary outcomes included HIV and HCV prevalence, and HCV test acceptance, refusal, risk, and knowledge.

**Results:**

We found no significant difference in HIV test acceptance between the bundled HCV/HIV (91.8%) and HIV-only (90.6%) groups (p=0.642). There were also no significant differences in test acceptance based on gender, race, or ethnicity. A majority of participants (76.6%) reported at least one HCV risk factor. No participants tested positive for HIV, and one (0.5%) tested positive for HCV.

**Conclusion:**

Integrating bundled, rapid HCV/HIV testing into an established HIV testing program did not significantly impact HIV test acceptance. Future screening efforts for HCV could be integrated into current HIV testing models to target high-risk cohorts.

## INTRODUCTION

The Centers for Disease Control and Prevention (CDC) estimates that 1.2 million individuals in the United States (U.S.) are living with human immunodeficiency (HIV) and 3.5 million individuals are living with hepatitis C virus (HCV).^1^–^4^ Due to similarity in risk factors and transmission, the prevalence of HIV/HCV co-infection is high. An estimated 25% of individuals living with HIV in the U.S. are co-infected with HCV, and approximately 80% of people with HIV who inject drugs also have HCV.^5^ Co-infection increases non-AIDS related morbidity and mortality in HIV-positive patients, more than tripling the risk for liver disease, liver failure, and liver-related death.^5^

The CDC recommends HCV testing for high-risk groups, including people who inject drugs (PWID), recipients of organ transplants or blood products prior to 1992, healthcare or public health workers exposed to HCV-infected blood, and one-time testing of all persons born between 1945 and 1965, a cohort accounting for 75% of all chronic HCV infections in the U.S.^6^–^8^ To augment screening, as of 2013 a New York State law mandates inpatient hospital and primary care settings to offer HCV tests to every individual in this birth cohort.^9^ However, reliance on risk-based testing may miss undiagnosed HCV-positive patients. Approximately 80% of infected individuals are asymptomatic, rendering diagnosis challenging without routine screening.^10^ Of those already infected, an estimated 50% are tested for HCV, about 43% enter into care, and only 9% achieve sustained viral response.^10^ In settings of high HCV prevalence, routine screening and counseling with prevention messages may facilitate earlier diagnosis, linkage to care, and transmission reduction.^11^

The emergency department (ED) is an ideal setting to increase access to routine screening and counseling services, particularly for high-risk populations that are less likely to have access to ongoing primary care.^12^–^14^ Immigrants, substance users, uninsured, and individuals with unstable housing situations often rely on EDs for incident and routine health care. These populations are also at higher risk of HCV and HIV infection, rendering the ED an important location to improve widespread healthcare delivery.

The high prevalence of HIV and HCV co-infection, similarity in testing strategies, and interrelated risk factors suggest a practical overlap in integrating screening services. A previous survey of patients during an ED or pharmacy visit found that more than half of the participants prefer hepatitis B/C testing to be in conjunction with HIV testing, rather than hepatitis alone.^15^ This integration could effectively use existing resources and infrastructure to address both epidemics and facilitate the linkage of HCV-infected individuals to care. Integrating HCV testing into existing HIV testing and counseling programs may also reinforce prevention education messages to reduce risky behavior among high-risk populations, particularly PWID.^16^

The objective of this study was to integrate rapid HCV testing into an established HIV testing and counseling program to evaluate the effect of rapid, bundled screening on HIV-test acceptance rate. Secondary outcomes include HCV test acceptance, identification of newly diagnosed HCV and HIV positive patients, HCV knowledge, risk assessment, and refusal reasons.

## METHODS

### Study Design

We obtained institutional review board (IRB) approval through the Albert Einstein College of Medicine IRB (IRB #2012-491, approved August 13, 2012). A two-armed, randomized controlled trial (RCT) was conducted at Jacobi Medical Center, a Level 1 trauma and tertiary-care center located in the Bronx, New York. The ED HIV screening program ran 24 hours a day seven days a week; however, screening was limited to the times when a trained research associate (RA) was available. In the six-month study period, an RA was available 75 days to screen and enroll patients. The hours of the RCT were limited to weekdays from 9am–5pm on these days. Upon recruitment, all participants completed questionnaires that included demographic information, HCV risk assessment, and HCV knowledge questions. Participants were randomized either to the control arm or the intervention arm. The control arm was offered HIV testing only, and the intervention arm was offered HIV testing concurrently with HCV testing (bundled HIV/HCV screening). The research protocol received approval from Albert Einstein College of Medicine IRB.

**Population Health Research Capsule**

What do we already know about this issue?

HIV testing in emergency departments (EDs) has significantly influenced the number of undiagnosed HIV infections. Similar ED screening efforts are now being applied to hepatitis C (HCV).

What was the research question?

We sought to determine the effect of integrating rapid HCV testing into an established HIV testing program.

What was the major finding of the study?

We found that offering rapid HCV tests in conjunction with rapid HIV tests did not adversely affect HIV test acceptance.

How does this improve population health?

Bundling HIV and HCV testing into a single screening program could improve population health by identifying and linking infected individuals to care.

### Sample Recruitment

Patients were recruited from the adult ED at Jacobi Medical Center. Recruitment took place during a six-month period from December 2012 to May 2013. Inclusion criteria required patients to be 18 years of age or older and to speak English or Spanish. Patients were excluded from the study if they were medically unstable as determined by their ED provider, unable to consent, did not speak Spanish or English, were known to be HIV and/or HCV positive, or had been tested for HIV/HCV in the prior six months. Patients who refused to participate in the study completed a short, anonymous refusal form, which captured demographic information and reason for refusal. There was no racial or gender bias in selection of participants. As noted above, we excluded non-English or Spanish-speaking patients based on our IRB policy that requires informed consent documents be available in the patient’s native language. These translated documents were not available for this study.

### Study Procedure

RAs were trained as public health advocates to perform HIV and HCV testing and counseling. They approached eligible patients in the ED and followed a script in asking patients if they were interested in participating in a study through which they would be offered free screenings recommended for their general health. Patients who refused the offer of the HIV and/or HCV tests completed a test-refusal questionnaire. All enrolled participants completed a questionnaire including demographic information, HCV risk assessment, and HCV knowledge. After providing verbal consent, participants were randomized to either an HIV test only group (control) or a bundled HIV/HCV test group (intervention). An independent statistician used a computer-generated allocation sequence to determine randomization. Randomization assignments were placed in sealed, opaque envelopes that were opened sequentially after verbal consent was obtained for the study.

Those randomized to the control group were offered only an HIV test, and those randomized to the intervention group were offered both HCV and HIV tests. The OraQuick® HCV rapid antibody test was employed as a rapid blood fingerstick test for HCV antibodies. The OraQuick *Advance*® rapid HIV-1/2 antibody test was used to test for HIV-1 and HIV-2 antibodies in oral fluid. Both point-of-care tests provide results in 20 minutes. Study subjects were not billed for either HIV or HCV testing.

A public health advocate delivered the test(s) results to the patient and conducted post-test counseling. In the case of a preliminary positive result on either test, the public health advocate informed the patient and the patient’s provider and scheduled a follow-up appointment for the patient. Patients who tested positive for HIV were linked to care according to the protocol already established by the existing HIV testing program.^14^ Patients who tested positive for HCV were similarly linked to care with a provider at the Adult Comprehensive Services clinic within Jacobi Medical Center, where blood was drawn for viral-load confirmatory testing. A public health advocate confirmed contact information for any positive patients to schedule follow-up appointments if necessary. After participants completed the study, educational materials from the CDC were provided on HIV and HCV.

### Outcome Measures

We compared the primary outcome, HIV test acceptance, between the control (HIV only) and intervention group (HIV and HCV). HIV test acceptance was used as an outcome proxy to evaluate the feasibility of integrating HCV testing into the established HIV testing program without adversely impacting HIV testing. Secondary outcomes included HIV and HCV incidence, HCV test acceptance, refusal reasons, risk level, and knowledge. We adapted the seven-question knowledge form from patient information sheets distributed by the CDC and a previously-validated hepatitis knowledge measure published in 2009.^5^ We identified characteristics associated with HCV test acceptance and HCV knowledge.

### Sample Size

We determined sample size using the following parameters: 1) 80% power; 2) significance level of 0.05; 3) two-sided significant test; and 4) 10% difference between groups on the acceptance of HIV testing. Using these parameters, a sample of 227 in each group was needed to test the primary outcome: acceptance of an integrated screening program for HIV and HCV infection. Groups of at least 333 were used to allow for drop-out and protocol violations.

### Statistical Analysis

Data was recorded in Microsoft Excel (Microsoft Corp., Redmond, Washington) throughout patient recruitment, from December 2012 to May 2013. Data obtained from subjects entered using unique subject numbers, without specific identifiers. This method of data management was used to ensure patient confidentiality.

We analyzed data using descriptive statistics to compare the baseline characteristics of study participants in the intervention and control groups. Categorical variables were compared with proportions and Fisher’s exact test-derived confidence intervals [CI]. Continuous variables were compared with means and 95% CIs for parametric data and medians for nonparametric data. We compared acceptance rates for HIV testing in experimental and intervention arms using chi-square test with Fisher’s exact test-derived CIs. Stata ®(StataCorp LLC, College Station, Texas) statistical software was used to tabulate participant demographics and testing frequencies for HIV, HCV, or both.

## RESULTS

Of the 733 patients assessed for study eligibility, 478 were eligible and agreed to participate (Figure). There were 244 participants in the control (HIV-only) arm and 234 participants in the experimental (bundled HCV/HIV) arm; 91.8% of the control arm accepted an HIV test and in the experimental arm, and 90.6% accepted an HIV test. We found no significant difference in HIV test acceptance between the HIV-only (90.6%, 212/244), and bundled HCV/HIV (91.8%, 224/234) groups (p=0.642). There were no significant differences in gender, race, ethnicity, or other participant demographics between the control and intervention groups (Table 1). Overall participant demographics were representative of the Bronx community; approximately 50% of participants were Hispanic, and approximately 40% were Black.

A total of 8.2% (20/244) of the control arm and 9.4% (22/234) of the experimental arm refused HIV testing (p-value 0.794) (Figure). More than half the participants refused HIV testing in each group because they did not feel they were at risk of contracting HIV (11/20 in the control arm and 15/22 in the experimental arm). Other reasons for refusals included the following: “I am afraid to find out my results;” “I don’t care whether I have HIV or not;” I don’t have time to test;” “I am worried that the test will slow my care;” “I am with family or friends;” or no reason given. None of the participants in either the control or experimental arm tested positive for HIV.

Acceptance of HCV testing was high in the bundled arm (79.9%, 187/234). The two main HCV test refusal reasons were “I do not want to have my finger stuck,” (29/47) and “I don’t feel that I am at risk of having hepatitis C” (23/47). Other refusal reasons included “I don’t have time to test,” “I don’t care whether I have hepatitis C or not,” “I am worried that the test will slow my care,” and no reason given. One (0.5%) participant in the experimental arm tested positive for HCV. A majority of participants (76.6%,) reported at least one HCV risk factor (Table 2). The most common risk factor reported was a tattoo (67.5%), followed by a piercing other than the ear (44.5%) and being a member of the birth cohort (1945–1965) (26.5%). Few participants reported ever (2.2%) or currently (1.6%) using injection drugs.

All study participants answered a hepatitis C knowledge questionnaire. A majority of study participants (74.3%) acknowledged that HCV-infected people can live for years with unrecognized infection. A total of 70.7% of participants responded that they knew that alcohol could damage the livers of people living with HCV, 66.9% knew that HCV can be transmitted sexually, 55.9% knew that HCV can be treated, 45.8% knew that HCV can be cured, and 43.9% knew that there was no vaccine available for HCV (Table 3). A total of 47.3% of patients knew that HCV infections are more common in people born between 1945 and 1965.

## DISCUSSION

This study is one of the first to implement an on-site, bundled, rapid HIV/HCV testing and counseling program in a high-volume urban ED. Integration of rapid HCV testing into a pre-existing HIV testing program did not adversely impact patients’ HIV test acceptance. These results indicate the feasibility of integrating HCV testing and counseling into established HIV testing programs as effective screening interventions to target high-risk populations.

For a mobile patient population – many with current or history of IDU, homelessness, or incarceration – a public health approach is indicated to counter structural barriers inhibiting the HCV care continuum.^17^,^18^ Impediments to timely diagnosis and care often include patients’ limited access to care, prohibitive costs, and insufficient provider training or incentive to screen and treat HCV infection. Incorporating point-of-care HCV testing and counseling into existing HIV screening infrastructure can counter these barriers by relying on public health advocates already trained to navigate patients to care in both clinical and non-clinical settings.

Findings are consistent with other studies that found that health counselors and patients are receptive to the incorporation of HCV counseling and testing into existing HIV screening programs.^19^ A previous study comparing the acceptance of HCV tests in different settings (i.e., correctional facilities, drug treatment facilities, field/visit outreach sites, HIV counseling/testing sites, sexually transmitted disease clinics, family planning clinics, and primary healthcare facilities) found the largest number of HCV tests were administered at HIV testing/counseling sites.^19^

However, the HCV diagnosis rate in this sample was lower than anticipated; only one participant tested positive for HCV (0.5%). This finding is inconsistent with previous studies evaluating bundled HCV/HIV screening. In the aforementioned study comparing HCV test acceptance and diagnosis rate across sites, almost 20% of participants who tested for HCV at HIV testing/counseling sites were HCV positive, suggesting that targeting HIV testing sites captures a population at high risk for HCV.^19^ In our study sample, the low prevalence of HCV is likely attributable to two factors specific to this study population – age and prior IDU. Very few of the individuals enrolled in the study fell within the birth cohort and only 2% had ever used injection drugs. Given that these two characteristics are significant predictors of HCV infection, it could explain why the HCV prevalence rate in this study was low. Furthermore, this was a convenience sample of a much larger ED population and the HCV prevalence rate in this study cannot be generalized to the larger ED population. The purpose of the study was to show that non-targeted HCV screening can be easily incorporated into existing HIV screening programs without any negative impact. This study was not meant or powered to characterize the overall HCV prevalence rate in the more general ED population.

Studies assessing HIV and HCV testing strategies for PWIDs have postulated that bundled HIV and HCV screening can lead to increased health testing rates and improved access to prevention and care.^16^ While previous studies evaluated HIV/HCV testing specifically in PWIDs, this study is one of the first to assess the impact of rapid, bundled, routine screening on an undifferentiated population in an urban ED.^16^,^20^ The ED has become an important setting to implement public health interventions for other infectious diseases, particularly HIV infection, to capture those lacking consistent access to primary care. Urban EDs have become a primary point of care for high-risk populations such as PWID, the unstably housed, undocumented immigrants, and the formerly incarcerated, contributing to the high prevalence of HCV in an ED setting.^12^,^13^,^21^

This study also demonstrated the feasibility of rapid HCV testing. Although conventional HCV testing is most commonly employed, this testing method requires extensive follow-up that is often challenging for high-risk populations, including homeless individuals, undocumented immigrants, and formerly incarcerated individuals.^22^ The enzyme immunoassay requires phlebotomy, which limits testing to clinical settings; it also poses additional challenges of finding a usable vein for PWID.

For high-volume settings such as the ED, rapid testing offers an effective intervention to diagnose and link high-risk patients for whom follow-up is not always feasible. Rapid HIV screening has been widely accepted as an efficient approach to identify and link to care HIV-positive individuals within an urban ED.^14^ Rapid HCV screening similarly addresses barriers previously inhibiting stages of the HCV care continuum by facilitating on-site delivery of results, counseling, and linkage to care.^22^ The OraQuick® HCV assay can detect HCV antibodies in oral fluid or blood and allows result delivery after 20 minutes, preventing the rampant loss of follow-up that occurs with conventional testing, while also allowing for screening in nonclinical settings.^23^ The accessibility of rapid testing for both HCV and HIV allows ease of integration of these point-of-care screening programs to maximize diagnosis and linkage to care.

## LIMITATIONS

The feasibility of integrating HIV and HCV screening services in this study relied heavily on the already well-established HIV-testing program at Jacobi Medical Center where the HIV test acceptance has been higher than reported in other studies.^14^ Outside the context of this RCT, approximately 85% of patients approached accept HIV testing.^14^ This unique model was designed specifically to address the testing and counseling needs of a high-risk population relying on the ED for healthcare needs and has been proven effective at providing quality education and screening services in this setting.^14^ Because of this unique pre-existing model, it is uncertain whether bundled, rapid HIV/HCV screening can be replicated with similar ease and efficiency in other settings. As a single-center study, the generalizability of these results to other hospitals or healthcare settings is uncertain. Additionally, the study was limited to individuals who spoke English or Spanish, limiting generalizability to other populations.

This sample also consisted of a negligible percentage of Asian participants, which is generally representative of the community this hospital serves in the Bronx, New York. However, recent surveillance data suggests that Asian and Pacific Islander populations are disproportionately affected by HCV, correlating with presence of tattoos, use of acupuncture needles, and IDU.^4^ While this study was intended to target the particular demographics of the inner-city borough, this limitation restricts generalizability to broader populations.

With the high rate of refusals to participate in the study (233 refusals), it is also possible that sampling bias impacts the generalizability of the study. The percentage of participants within the high-risk birth cohort was lower than anticipated in this sample (26.5%), and it is uncertain whether more patients within the birth cohort would be as receptive. Self-perceived low risk was the most common reason for refusing screening, both within the HIV-only arm and the bundled HIV/HCV arm. However, the HCV knowledge survey indicated suboptimal understanding of the increased risk among the birth cohort (47.3% correct). Future public health interventions should include educational components to increase awareness of risk factors. Those who refused HCV testing also commonly reported, “I do not want to have my finger stuck.” It is likely that rapid testing with the more recently developed OraQuick® oral swab would reduce refusals in future screening interventions.

## CONCLUSION

This study demonstrated that integrating rapid HCV and HIV testing is an effective and efficient approach to screen at-risk populations in an urban ED setting. Offering rapid HCV tests in conjunction with rapid HIV tests did not adversely affect HIV test acceptance; both HIV and HCV test acceptance rates were high. Both the high prevalence of patient risk factors and suboptimal HCV knowledge underscore the need to implement and sustain rapid HCV testing. Further studies should evaluate the feasibility of establishing new bundled HIV/HCV screening programs where rapid testing infrastructure does not yet exist.

## Figures and Tables

**Figure:**
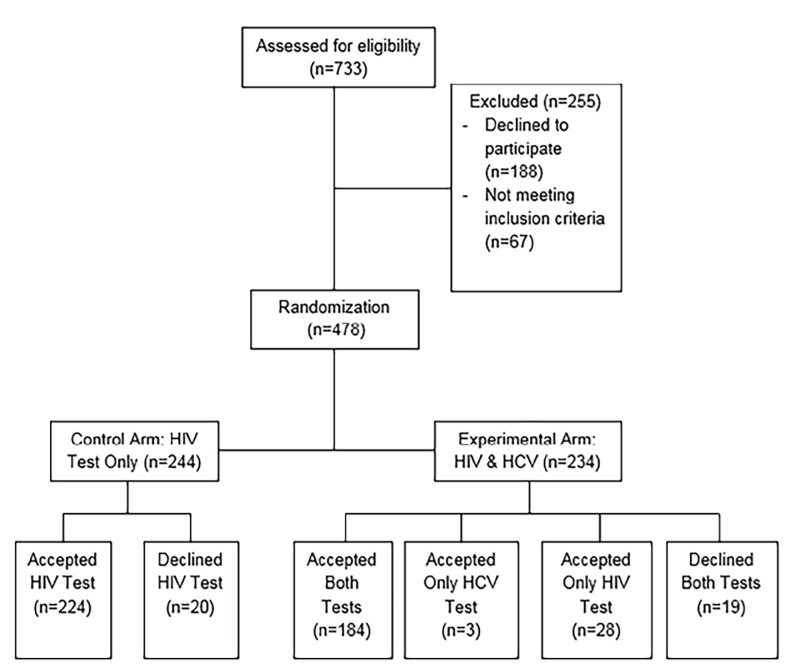
CONSORT Diagram: Participants enrolled in a randomized controlled trial to evaluate integration of HCV testing into a rapid HIV testing program. *HIV,* human immunodeficiency virus; *HCV,* hepatitis C virus.

**Table 1:** Participant demographics of a randomized controlled trial to evaluate feasibility and efficacy of bundled HIV/HCV rapid testing.

Demographics	Control arm: HIV Only (n=244)	Experimental arm: HIV & HCV (n=234)
Age	35.8±13.5	35.5 ±13.0
Gender		
Male	43.4%	45.3%
Female	55.7%	53.8%
Transgender	0.8%	0.4%
Ethnicity		
Hispanic	52.9%	53.8%
Non-Hispanic	45.9%	44.9%
Race		
Black	39.3%	36.3%
White	12.7%	13.7%
Other	34.5%	36.7%
Education		
0–8th grade	7.0%	8.1%
Some high school	20.5%	20.1%
High school degree	58.7%	58.6%
College degree	7.8%	7.3%
Graduate degree	0.8%	1.7%
Insurance		
Medicaid	32.0%	37.6%
Medicare	4.5%	2.6%
Private	23.4%	20.1%
Not insured	35.7%	35.0%
Previously tested for HIV	89.0%	89.7%
Previously tested for HCV	37.7%	38.0%

*HIV,* human immunodeficiency virus; *HCV,* hepatitis C virus.

**Table 2:** Reported hepatitis C virus risk factor prevalence in urban emergency department patient cohort.

Risk factors	Percentage
Tattoo	67.5%
Piercing other than the ear	44.5%
Birth cohort (1945–1965)	26.5%
Sex with someone who exchanged sex for money or drugs	12.3%
Accidental needle stick at work	9.6%
Lived with someone who is HCV positive	5.2%
Sex with a PWID	4.6%
Blood transfusion or organ transplant before 1992	3.3%
Sex with MSM	3.3%
Sex with someone who is HCV positive	3.0%
Ever used injection drugs	2.2%
Currently using injection drugs	1.6%
Long term dialysis	1.4%
Ever used methamphetamine (crystal meth)	1.4%
Received blood clotting factor before 1987	3.3%

*HCV,* hepatitis C virus; *PWID,* person who injects drugs; *MSM,* men who have sex with men.

**Table 3:** Hepatitis C virus knowledge questions and percentage correct responses from a patient cohort of an urban emergency department.

7-Question true/false knowledge measure (n=478)	% Correct
Hepatitis C can be given to someone during sexual intercourse. (T)	66.9%
There are no treatments for hepatitis C. (F)	55.9%
People can live with hepatitis C for many years without knowing that they have been infected with the virus. (T)	74.3%
People living with hepatitis C can damage their liver if they drink alcohol. (T)	70.7%
There exists a hepatitis C vaccine that can be used to prevent people from getting infected with the hepatitis C virus. (F)	43.9%
There is no cure for hepatitis C. (F)	45.8%
Hepatitis C infections are more common in people born between 1945 and 1965. (T)	47.3%
